# Combination Therapy With Risperidone and High-Dose Melatonin Is Effective Despite Reversible Side Effects Including Breast Budding

**DOI:** 10.7759/cureus.74607

**Published:** 2024-11-27

**Authors:** Akihiro Nakamura

**Affiliations:** 1 Pediatrics, Child and Parents Support Clinic, Ebina, JPN

**Keywords:** breast budding, melatonin, prolactin, restlessness, risperidone, sleep disorders

## Abstract

Melatonin is commonly used to treat sleep disorders. Regardless of the prolactin level elevation induced by melatonin administration, breast budding is not known to develop as a result of this treatment. A 10-year-old boy presented to our outpatient clinic with restlessness and sleep disorders. Risperidone (0.5 mg/day) and melatonin (1 mg/day) were orally administered. His daytime concentration improved after increasing the melatonin dosage to 2, 3, and 4 mg/day every 2 weeks, although his nighttime awakenings did not completely improve. After continuing high-dose melatonin treatment for one month, the patient experienced pain in his left mammary gland and developed breast budding. However, it disappeared promptly after the discontinuation of melatonin. Risperidone and high-dose melatonin administration effectively reduced restlessness. Administration of risperidone and high-dose melatonin may cause breast budding; however, breast budding is reversible upon the discontinuation of melatonin, and we suggest that clinicians prescribe medication as needed to improve these symptoms.

## Introduction

Autism spectrum disorders (ASD) are not rare; many primary-care pediatricians care for several children with ASD. In fact, a survey completed in 2004 revealed that 44% of pediatricians reported that they care for at least 10 children with ASD [1]. Malow et al. investigated the prevalence of sleep disorders in 1,518 children aged 4 to 10 years diagnosed with ASD and enrolled in the Autism Speaks Autism Treatment Network Registry [2]. The investigators found that 71% of the children experienced sleep disorders, although only 30% had been diagnosed with a sleep disorder [2].

Melatonin is commonly used to treat sleep disorders [3]. The dose range in Japan is 1-4 mg/day. Melatonin is a safe and well-tolerated medication in children and adolescents. A double-blind study lasting 39 weeks involving 95 subjects who received melatonin (2-5 mg) indicated that the common side effects of melatonin include fatigue (18.9%), vomiting (16.8%), somnolence (16.8%), cough (13.7%), mood swings (13.7%), and upper respiratory infection (10.5%) [3,4]. The mean age was 9; the range was 2-17.5 years; 74.7% were males [4]. However, there was no report of breast budding.

Gynecomastia is prevalent among adolescent men, and approximately 25% of gynecomastia cases are due to natural hormonal changes during puberty and are self-limiting [5]. True gynecomastia is typically associated with excess estrogen production, and a certain number of cases are drug-induced [5]. It usually occurs bilaterally and symmetrically and is the most common breast condition in men [6]. Hyperprolactinemia is known to be a common side effect in males 10-20 years old diagnosed with ASD or disruptive behavior treated over the long term with risperidone [7]; however, there was no report of breast budding by risperidone or melatonin, and the association between breast budding and drug use remains unknown. On the other hand, Liat A et al. also reported that a combination of risperidone and melatonin improved circadian rhythm in sleep disorders [8]. Herein, we report the case of a 10-year-old Japanese boy with ASD who was diagnosed with sleep disorders.

## Case presentation

A 10-year-old boy presented to our outpatient clinic with restlessness and sleep disturbances. The patient’s medical and family history were unremarkable. Although the patient’s grades at school were average, he could not remain seated during class, play collaboratively, or perform activities independently. He was proficient in performing calculations and writing words but struggled with reading sentences. Additionally, he was not allowed to enter certain rooms, such as music classrooms, two years prior to presentation due to communication disorders. As a result, he had difficulty sharing interests and feelings with his classmates. His communication was disorganized, and he showed no interest in his peers. Furthermore, he became overly sensitive to noise and attached to certain classmates. He impulsively left regular classes and became increasingly isolated. Consequently, the patient was diagnosed with ASD using the fifth edition of the Diagnostic and Statistical Manual of Mental Disorders (DSM-5) [9]. Oral risperidone (0.5 mg/day) and melatonin (1 mg/day) were administered for two weeks.

Following treatment, the patient’s isolated behavior improved quickly from the perspective of his elementary school teachers, and he was able to concentrate in class; however, his nighttime awakenings did not completely improve. His daytime concentration further improved after gradually increasing the melatonin dosage to 2, 3, and 4 mg/day every two weeks, although nighttime awakenings persisted. Therefore, we suggested that the patient and his family increase his daytime activity, as we speculated that low activity during the day was a contributing factor to his sleep disturbances.

**Figure 1:**
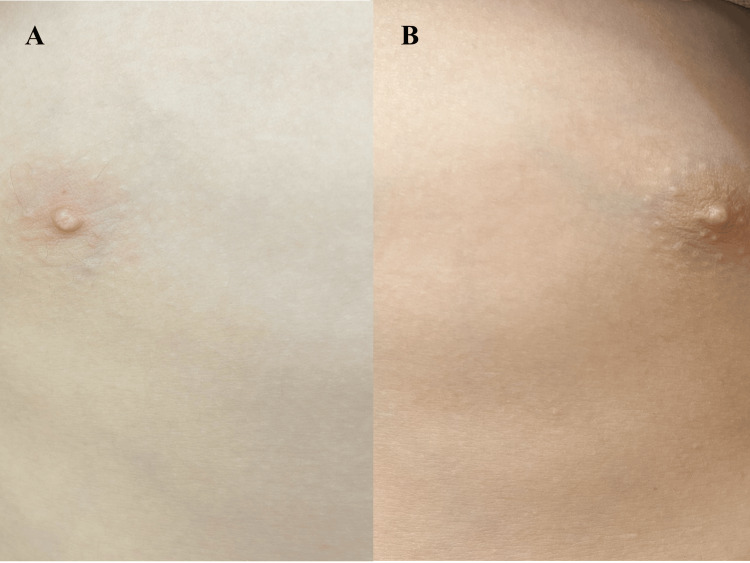
Images showing left-sided breast budding (1.5 × 2.0 cm). A) Image showing the normal right side of the chest. B) Image showing left-sided breast budding (1.5 × 2.0 cm).

After continuing the melatonin dosage at 4 mg/day for one month, he experienced pain in his left mammary gland and developed breast budding (1.5 cm × 2.0 cm) (Figures 1a-1b).

Initially, the patient’s mother took him to the breast department outpatient clinic, where the breast surgeon suggested discontinuing melatonin. Subsequently, they consulted us, and we decided to continue low-dose risperidone therapy after discontinuing melatonin, as high-dose melatonin administration had not been fully effective for his sleep disturbances. Simultaneously, we conducted blood tests, including assessments of liver function, renal function, thyroid hormones, and gonadal hormones (Table 1).

**Table 1:** Blood sample test results at the time the patient noticed breast budding. CBC: Complete blood count; Hb: Hemoglobin; Ht: Hematocrit; PLT: Platelet; T-P: Total protein; T-Bil: Total bilirubin; AST: Aspartate aminotransferase; ALT: Alanine aminotransferase; BUN: Blood urea nitrogen; Cre: Creatinine; TSH: Thyroid-stimulating hormone; Free-T3: Free triiodothyronine; Free-T4: Free thyroxine; LH: Luteinizing hormone; FSH: Follicle-stimulating hormone.

CBC	Results	Reference range
WBC	7800 /μL	3300-9000 /μL
RBC	466 ×10^4 ^/μL	430-570 ×10^4 ^/μL
Hb	13.1 g/dL	13.5-17.5 g/dL
Ht	38.7 %	39.7-52.4 %
PLT	35.8 × 10^4 ^/μL	14-34 × 10^4 ^/μL

Biochemistry		
T-P	7.3 g/dL	6.7-8.3 g/dL
T-Bil	0.5 mg/dL	0.2-1.2 mg/dL
AST	21 U/L	10-40 U/L
ALT	9 U/L	5-45 U/L
BUN	17.5 mg/dL	8.0-20.0 mg/dL
Cre	0.51 mg/dL	0.61-1.04 mg/dL
Na	141 mg/dL	137-147 mg/dL
K	4.7 mg/dL	3.5-5.0 mg/dL
Ca	9.5 mg/dL	8.4-10.4 mg/dL
P	4.7 mg/dL	2.5-4.5 mg/dL

Endocrinological values		
TSH	1.44 mIU/L	0.350-4.94 mIU/L
Free-T3	3.79 ng/dL	1.68-3.67 ng/dL
Free-T4	1.09 pg/mL	0.70-1.48 pg/mL
LH	0.35 mIU/mL	0.79-5.72 mIU/mL
FSH	2.11 mIU/mL	2.00-8.30 mIU/mL
Estradiol	<10 pg/mL	19-51 pg/mL
Progesterone	0.1 ng/mL	<0.6 ng/mL
Testosterone	0.19 ng/mL	1.92-8.84 ng/mL
Prolactin	22.58 ng/mL	1.1-9.5 ng/mL

The results showed that his prolactin level was slightly elevated (≧20 ng/ml), while his estrogen and testosterone levels were below the detectable range. Breast budding disappeared within two days. The patient had equally sized testes (2.5 cm × 3 cm) and no pubic hair. According to the Tanner classification, the condition was classified as stage 2, and G2P1 [10].

Risperidone was discontinued one month after discontinuing melatonin because the patient was able to play baseball collaboratively and remain in the classroom. Consequently, his sleep disturbances did not worsen. His restlessness and impulsive behaviors disappeared, and he began participating in group activities. His parents reported that his range of movement and amount of exercise had expanded, and he was no longer waking in the middle of the night after discontinuing both medications.

Blood tests were repeated three months after discontinuing melatonin and two months after discontinuing risperidone. The results confirmed that his prolactin levels had returned to normal (8.66 ng/ml), and his testosterone levels were slightly elevated (3.31 ng/ml).

## Discussion

To the best of our knowledge, this is the first reported case of breast budding induced by the combination of risperidone and high-dose melatonin. Gynecomastia is defined as the benign proliferation of glandular breast tissue in men [11]. The primary treatment involves discontinuing contributing medications and addressing underlying diseases. Gynecomastia occurs when the estrogen-to-testosterone ratio in men is disrupted [5]. However, in this patient, estrogen and testosterone levels were below detectable limits when breast budding was observed (Table 1).

In addition, breast scans did not show the typical bilateral gynecomastia (Figures 1a-1b). Therefore, we concluded that this phenomenon represented breast budding.

In general, high prolactin levels (≧20 ng/ml) are uncommon in patients with gynecomastia [5]. Both risperidone and melatonin are known to induce hyperprolactinemia, but no studies have reported adverse events associated with melatonin administration [12]. However, these studies primarily evaluated melatonin use in adults over short periods. In the future, breast budding may be recognized as a possible side effect of prolonged administration of risperidone and high-dose melatonin, as the condition improved within a few days after discontinuing melatonin alone.

Considering the patient’s age, luteinizing hormone (LH), and testosterone levels, the episode was likely associated with physiological breast budding, which is frequently observed in the early stages of male puberty. Nevertheless, we suggest that elevated prolactin levels may have contributed to breast budding [13].

Notably, sexual maturation was delayed in young male rats continuously administered melatonin from day 20 to day 115 of life [14]. Investigators discovered that melatonin’s inhibitory effects were most significant between days 20 and 30 but were reversible when melatonin administration ceased after 45 days [14].

In our patient, testosterone levels were below detectable limits 60 days after initiating oral melatonin (Table 1). These suppressed testosterone levels were attributed to melatonin use. However, testicular enlargement was appropriate for the patient’s age, indicating that testosterone levels increased after discontinuing melatonin and that testicular size was not affected by the treatment.

The administration of risperidone and high-dose melatonin was effective in managing restlessness. According to product documentation, the recommended melatonin dose range in Japan is 1-4 mg/day. Typically, 1-2 mg/day is effective in infants, although the required dose tends to increase with age. High-dose melatonin appeared to be partially effective in this adolescent. In this case, the combination therapy improved classroom concentration and provided partial relief from sleep disorders. Similarly, Liat A et al. reported that combining risperidone and melatonin improved circadian rhythm in sleep [8]. Therefore, we recommended that the patient and his family increase daytime activity as part of behavioral therapy. As a result, the patient began playing baseball every weekend and no longer felt nervous around others. We speculate that changes in daytime activity contributed to improved sleep, as high-dose melatonin alone did not fully resolve sleep disturbances. Mental development with age may also have played a role in the success of behavioral therapy, as juvenile patients often experience recurring restlessness and sleep disorders after discontinuing risperidone and melatonin.

Breast budding is reversible upon the discontinuation of melatonin, and we suggest that clinicians prescribe medications as needed to manage these symptoms.

## Conclusions

The combination of risperidone and high-dose melatonin administration may cause breast budding, as both are known to induce hyperprolactinemia. Additionally, the patient's testosterone levels were suppressed during melatonin administration. However, the breast budding and suppressed testosterone levels resolved after discontinuing melatonin. As a result of risperidone and high-dose melatonin administration, restlessness and sleep disorders improved. Notably, these symptoms did not recur even after the discontinuation of drug therapy. While risperidone and high-dose melatonin were effective in improving restlessness and sleep disorders, potential side effects, such as reversible breast budding, warrant careful monitoring and cautious use. 

Further research is required to better understand the potential side effects of combining risperidone, typically used for schizophrenia, with melatonin for sleep disorders, as this combination may induce side effects like breast budding.
